# Accurate genome-wide predictions of spatio-temporal gene expression during embryonic development

**DOI:** 10.1371/journal.pgen.1008382

**Published:** 2019-09-25

**Authors:** Jian Zhou, Ignacio E. Schor, Victoria Yao, Chandra L. Theesfeld, Raquel Marco-Ferreres, Alicja Tadych, Eileen E. M. Furlong, Olga G. Troyanskaya

**Affiliations:** 1 Lewis-Sigler Institute for Integrative Genomics, Princeton University, Princeton, New Jersey, United States of America; 2 Graduate Program in Quantitative and Computational Biology, Princeton University, Princeton, New Jersey, United States of America; 3 Center for Computational Biology, Flatiron Institute, New York, New York, United States of America; 4 Genome Biology Unit, European Molecular Biology Laboratory (EMBL), Heidelberg, Germany; 5 Department of Computer Science, Princeton University, Princeton, New Jersey, United States of America; New York University, UNITED STATES

## Abstract

Comprehensive information on the timing and location of gene expression is fundamental to our understanding of embryonic development and tissue formation. While high-throughput *in situ* hybridization projects provide invaluable information about developmental gene expression patterns for model organisms like *Drosophila*, the output of these experiments is primarily qualitative, and a high proportion of protein coding genes and most non-coding genes lack any annotation. Accurate data-centric predictions of spatio-temporal gene expression will therefore complement current *in situ* hybridization efforts. Here, we applied a machine learning approach by training models on all public gene expression and chromatin data, even from whole-organism experiments, to provide genome-wide, quantitative spatio-temporal predictions for all genes. We developed structured in silico nano-dissection, a computational approach that predicts gene expression in >200 tissue-developmental stages. The algorithm integrates expression signals from a compendium of 6,378 genome-wide expression and chromatin profiling experiments in a cell lineage-aware fashion. We systematically evaluated our performance via cross-validation and experimentally confirmed 22 new predictions for four different embryonic tissues. The model also predicts complex, multi-tissue expression and developmental regulation with high accuracy. We further show the potential of applying these genome-wide predictions to extract tissue specificity signals from non-tissue-dissected experiments, and to prioritize tissues and stages for disease modeling. This resource, together with the exploratory tools are freely available at our webserver http://find.princeton.edu, which provides a valuable tool for a range of applications, from predicting spatio-temporal expression patterns to recognizing tissue signatures from differential gene expression profiles.

## Author summary

When and where a gene is expressed is fundamental information for understanding embryonic development. Current knowledge for such expression patterns is typically far from complete. Even for the long-standing model organism, *Drosophila melanogaster*, with large-scale *in situ* projects that have provided invaluable expression information for many genes, 40% of the genes still lack spatio-temporally resolved expression information. Such data is complemented by transcriptome datasets such as microarray and RNA-seq, which have whole-genome coverage and measure expression levels with greater dynamic range, but they typically lack precise spatio-temporal resolution. To bridge this gap, we developed a machine learning approach that combines the spatio-temporal resolution of *in situ* data with the accurate quantification and whole-genome coverage of genomic experiments, integrating information from 6,378 expression and chromatin profiling data sets. With this new approach, we present a genome-wide resource of spatio-temporal gene expression predictions for over 200 tissue-developmental stages during *Drosophila* embryogenesis. This resource is experimentally validated to have high-quality predictions, can guide the discovery of new tissue-specific genes, and provides a new tool to perform genome-wide analyses of spatio-temporal specificity.

## Introduction

Spatio-temporal gene expression information is fundamental for understanding the intricate mechanisms of embryonic development, which involves both precisely controlled differentiation into dozens of tissue types, as well as dramatic temporal transitions such as gastrulation and segmentation.

In the model organism *Drosophila melanogaster*, decade-long efforts in systematically characterizing developmental and tissue-specific expression patterns have greatly advanced the understanding of embryonic development and tissue formation [1–3]. Large-scale projects involving *in situ* hybridization and imaging, most representatively the Berkeley Drosophila Genome Project (BDGP) [1], have resulted in thousands of gene expression patterns now manually annotated to very specific tissues with controlled vocabularies [1]. These measurements render *Drosophila* embryonic development one of the most comprehensively characterized systems of spatio-temporal transcriptional regulation.

However, the current compendium of embryonic expression is not without limitations, even for this deeply investigated model organism. For many tissues only a small number of gene’s have been annotated to be expressed, with a median of 80 genes for each tissue, which is likely an underrepresentation of the true number of tissue-expressed genes. In addition, more than 4,000 protein-coding genes, as well as many non-coding genes, have not been annotated at all [1]. Moreover, the expression measurements *via* classical *in situ* hybridization and imaging are typically qualitative or grossly semi-quantitative [4]. Lastly, the limited dynamic range of standard *in situ* hybridization protocols that are typically used in large-scale efforts restricts the detection of lowly expressed genes.

To address these limitations, *in situ* data can be complemented by integrating them with the ever-growing transcriptome data measured by microarray or RNA-seq. With their whole-genome coverage and quantitative nature, the advantages of transcriptome profiles are highly complementary to those of *in situ* data. Even for cases where the transcriptome profiles are not tissue- or stage-specific, they are an information-rich source of co-regulated gene expression patterns, such as those of genes within the same regulatory or biochemical pathways. In these datasets, as gene expression is measured in a diversity of genetic or environmental conditions, functionally related genes are often co-expressed or perturbed together [5,6]. Using machine learning algorithms and known tissue-specific gene standards, these co-regulatory patterns can be extracted to infer tissue specific expression of genes, which combine advantages of both data types. Our previous work has shown that such an approach can accurately predict cell type-specific gene expression in the human kidney [6] and worm tissues [5] from non-tissue specific data, using manually curated cell type gene markers. In *D*. *melanogaster* embryonic development, the well-annotated resource of gene expression across tissues and developmental time, provide a unique opportunity to systematically predict not only tissue-specific expression but also the temporal stage of gene expression. Prior work has demonstrated the feasibility of making such predictions in a small set of 15 tissue-stages [7]. Moreover, considering the established tissue lineage information for *D*. *melanogaster* development [1], which has been curated into a tissue developmental ontology, the prediction algorithms can be further improved by integrating information across tissue-stages while considering the ontological relations between cell types.

Here, we present an approach for the prediction of gene expression during embryonic development in *D*. *melanogaster* based on integrating the tissue specificity of *in situ* hybridization data with the quantitative and comprehensive measurements from public genomic data compendium. The algorithm, which we refer to as structured in silico nano-dissection (SIND), improves individual tissue predictions with a cell lineage-aware prediction integration stage. This stage learns and applies transcriptional dependencies between developmentally and anatomically related tissue-stages, with lineage knowledge encoded in the structure of a probabilistic graphical model based on the tissue developmental ontology.

Using our SIND algorithm, together with *in situ* gene annotations and a compendium of 6,378 genome-wide expression and chromatin immunoprecipitation (ChIP) datasets, we predicted global expression patterns in 282 tissue-developmental stages, including >4000 genes without prior *in situ* data. The predictions were validated both computationally, *via* hold-out validation, and experimentally in the case of new predictions without prior literature evidence. We verified predictions in muscle and brain for 13 novel genes with no prior *in situ* data or literature evidence to the extent of our knowledge, for both tissue-specificity and temporal specificity, including co-expression patterns in multiple tissues, demonstrating the accuracy of our predictions. The complete and quantitative nature of the predictions enables previously infeasible analysis of tissue specificities, such as analyzing tissue-specific signatures in whole-animal differential expression datasets and mapping human diseases to *D*. *melanogaster* tissue/stages for aiding the development of human disease models in the fly. We provide all our predictions and exploratory tools in a user-friendly web interface FIND (fly in silico nano-dissection, find.princeton.edu) that allows the user to query predicted gene expression pattern and perform tissue-specificity prediction task.

## Results

### Integrating genomic data for embryonic tissue-developmental stage-specific gene expression prediction

**Fig 1:**
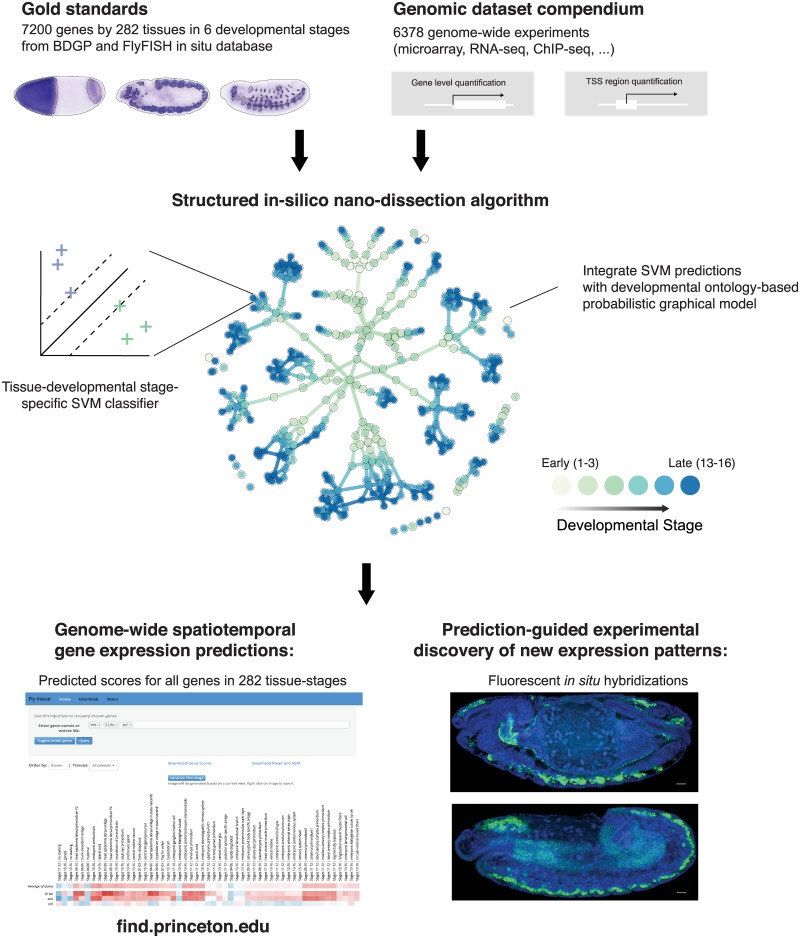
Schematic overview of *Drosophila melanogaster* spatial-temporal gene expression prediction and validation strategy. The abundance and diversity of high-throughput genomic data, and the spatio-developmental specificity of large-scale *in situ* experiments allow us to train machine learning models that predict gene expression spatial-temporal specificity in a genome-wide scale. We developed a developmental cell lineage-aware machine learning algorithm, structured in silico nano-dissection (SIND), for this task. First, we trained an array of SVM-perf classifiers for all tissue-stage categories. We then integrated and improved predictions for each category by utilizing tissue-stage relationships with a conditional random field model based on tissue developmental ontology. The validation of predictions was done with prior literature, hold-out in situ data, and new in situ experiments. We verified the spatio-developmental expression pattern of a number of proteins without prior knowledge on expression specificity.

As an overview (Fig 1, see also Methods), our approach first predicts gene expression probabilities for each tissue-stage term individually by learning linear models of transcription and chromatin signals that predict tissue-stage specific expression levels using a structural support vector machine (SVM) algorithm, and then integrates individual tissue-stage term predictions with a global developmental lineage-aware probabilistic graphical model. We named the *Drosophila* tissue-developmental stage gene expression prediction system FIND.

Conceptually, in the first stage, our method discovers and exploits the co-regulatory patterns of cell lineage-specific genes from high-throughput measurements of samples from diverse tissue origins and under a wide range of genetic, chemical, or environmental perturbations [5,6]. The cell lineage-specific genes were prioritized based on a weighted subset of conditions found to be informative by the algorithm. Notably, the high-throughput datasets we used as input do not have to be as tissue-specific as the predicted target tissue-stage, because we only require the co-regulatory patterns of tissue-specific genes to be shared across a subset of datasets [6]. In fact, most of the tissues we make predictions for are fine embryonic structures without existing high-throughput microarray or RNA-seq data specific for those structures.

In the second stage, we use prior developmental cell lineage knowledge to help integrate individual tissue-stage specific predictions with a conditional random field (CRF) probabilistic graphical model. Developmental lineage knowledge is expected to be informative for improving spatial-temporal predictions. This is both because gene expression in a precursor tissue usually provides informative insight into gene expression patterns of the tissue it develops into, and because related tissues, e.g. different cell types in the nervous system, are likely to share expression patterns for many genes. Specifically, we estimate the quantitative gene expression dependencies between developmentally related tissues empirically from *in situ* annotations and use it for integrating predictions. For each gene, the probabilistic graphical model treats expression in each tissue-stage term as a binary random variable. The value of each tissue-stage variable is dependent on both the input to the model, which are the SVM predictions from individual classifiers, and the values of other tissue-stage variables that are connected in the tissue developmental ontology. As the expression for each tissue is globally predicted considering dependencies between all tissue-stage terms, this approach allows for borrowing information from relevant tissues.

Spatio-temporal gene expression during *Drosophila* embryogenesis has been extensively studied via large-scale *in situ* hybridization projects, such as the BDGP *in situ* database [1] and Fly-FISH database [2]. These studies provide a very rich resource of spatio-temporal specificity of gene expression and are used as training standards for our models. Our genome-wide data integration approach complements the current *in situ* hybridization data in multiple ways: our approach makes predictions for plenty of genes (>4,000) unmeasured or undetected by *in situ*, computes quantitative scores comparable across genes and tissues and predict expression probabilities across all tissues, reducing the bias of manual annotation towards the most prominent or easy to recognize tissues.

**Fig 2:**
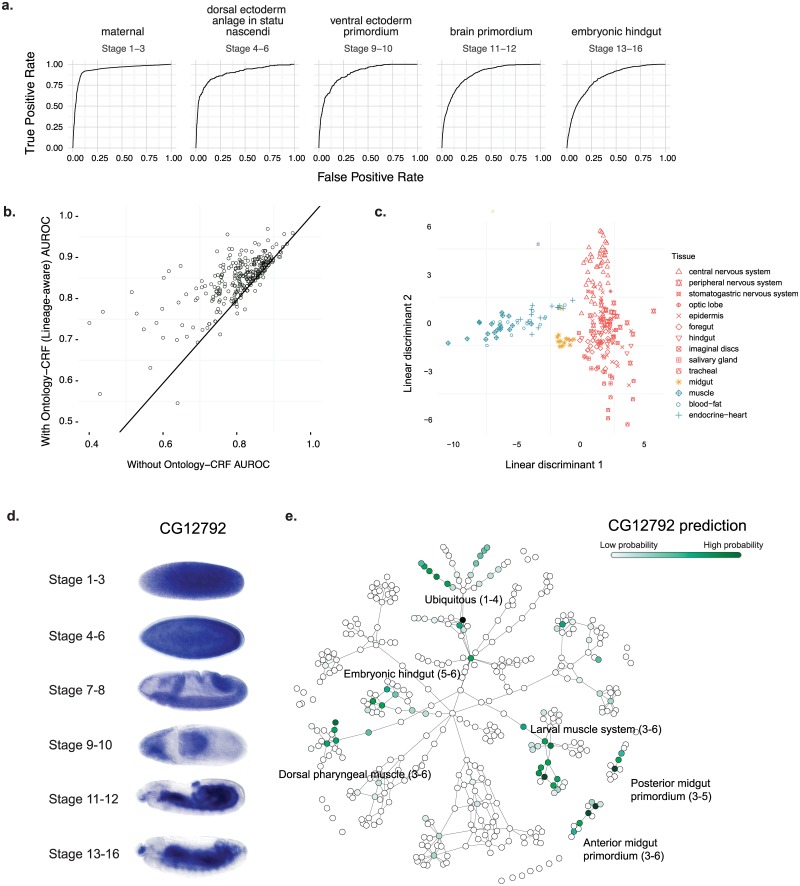
Tissue-developmental stage specificity of gene expression predictions. a. Gene expression prediction performances for selected tissue-stage categories measured by receiver-operating characteristics (ROC) curves. b. Integrating prediction across tissue-stages utilizing tissue developmental ontology information by ontology-CRF method improved prediction performances. The left panel compares area under ROC curves (AUROCs) for each tissue-stage category with and without using ontology-CRF integration. c. Global structure of predicted tissue-stage transcriptomes by embryonic layer of origin. The two dimensional projections of high-dimensional gene expression probability predictions were obtained with linear discriminant analysis (LDA) to best separate the tissue groups. Each point corresponds to a tissue-stage, the shape represent the tissue group and the color represent the embryonic layer of origin: light blue for mesoderm, yellow for endoderm and red for ectoderm. d-e. Example tissue-stage specificity predictions for CG12792. The observed expression patterns based on *in situ* hybridization were shown in d. The tissue-developmental categories and relationships were shown as a tree-like network (e), with nodes indicating tissue-stage categories and edges indicating “development from” and “part of” relationships. The color of each node represents the predicted probability of that category for the gene. Darker green color indicates higher predicted probability. Major tissue annotations and corresponding stages were labeled by text.

We first obtained a compendium of 6,378 high-throughput transcriptomic and chromatin profiling experiments, in addition to training standards consisting of *in situ* annotations for 282 tissue-stage terms spanning 6 time periods of fly embryonic development (outlined in S1 File). The developmental cell lineage information is encoded by a tissue developmental ontology based on an earlier work [6] (Supplementary File 1). We then used this data to train support vector machine classifiers and global integration model. We obtained a performance of median area under receiver-operating characteristics (AUROC) 0.87 across 282 tissue-stage terms (Fig 2a and 2b, S2 File). The predictions for each gene were made by five-fold cross-validation (see Methods).

Integrating individual tissue predictions with our conditional random field (CRF) model is a major factor for enhancing performance, in addition to utilizing a wide range of datasets and data types (S1 Fig). Using the ontology-based CRF method that we developed provides a consistent improvement across almost all tissues (Fig 2b), demonstrating the importance of the integrative approach allowing sharing information cross tissue-stages with developmental cell lineage. The global topological structure of the predicted tissue-stage-specific transcriptomes, as visualized by LDA and PCA, shows organization by both developmental stage order (S2 and S3a Figs) and by embryonic layer of origin (Fig 2c and S3b Fig).

### Specificity of embryonic tissue and developmental stage for individual genes predictions

For each gene, our predictions specify the temporal range of expression and the specific cell lineages at multiple time points in the developmental trajectory. This is demonstrated for *CG12792*, which is a ubiquitously expressed genes at early developmental stages, with more restricted expression at 13 hours after egg laying (AEL) (Fig 2d). This developmental trajectory is well captured by the cross-validated predictions when all expression labels for these genes were held out (Fig 2e). Based on our predictions, the probabilities for ubiquitous expression decrease during development, while the probabilities for specific cell lineages (muscle and midgut) increase over time. Importantly, these increasingly restricted expression patterns in those specific tissues are an accurate recapitulation of the expression dynamics detected by the *in situ* experiments (Fig 2d).

To systematically measure the performance of spatial and temporal pattern predictions, we compared the predictions of each gene to the expected tissues and temporal stages defined by *in situ* and to the tissue-stages with no detectable *in situ* expression. This is another important dimension of prediction performance, besides correctly ranking genes for each tissue. The distribution of AUROC for genes is shown in (S4 Fig). Even for genes with very complex expression patterns (> 20 annotations), an average AUROC of 0.89 is obtained (S4 Fig).

### Experimental and literature-based validation of predictions across embryonic tissues and developmental stages

We examined the ability of the model to predict expression patterns (outside of the training set) by both collecting gene expression information from the literature and performing fluorescent *in situ* hybridization (FISH) experiments for genes where no prior tissue-developmental mapping was available.

**Fig 3:**
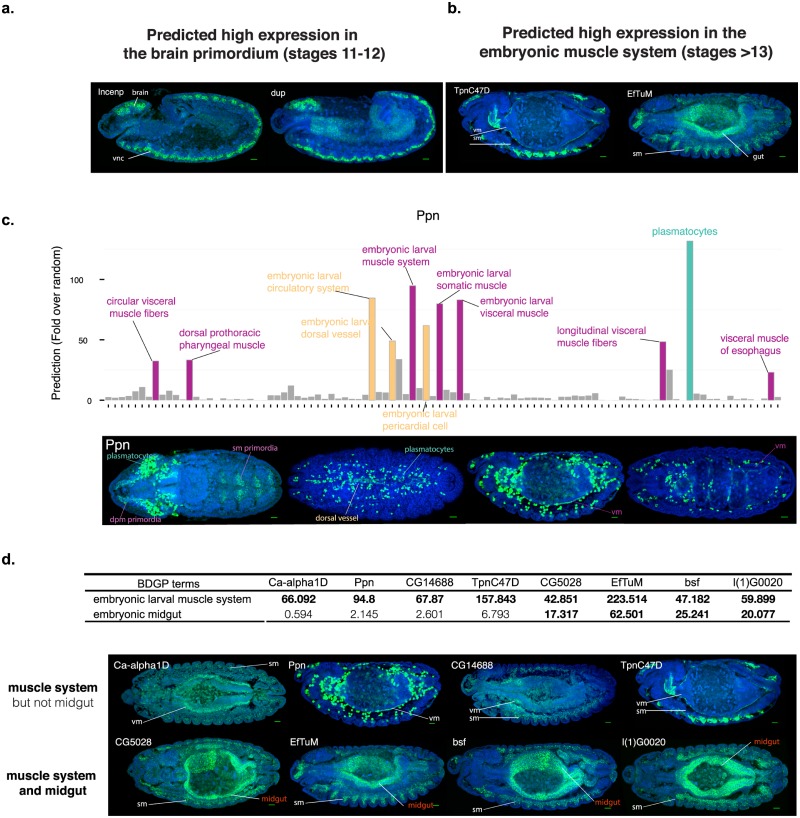
Experimental assessment of new expression pattern predictions. a. Two examples of genes tested by FISH in stage 12 embryos, showing expression in brain and ventral nerve cord (vnc). b. Two examples of genes tested by FISH showing expression in different muscle types. Embryos are stage 14 (*left*) and stage 13 (*right*). sm: somatic muscle, vm: visceral muscle. c. Accurate predictions of Ppn complex expression patterns in late embryo. *Top*. Predictions of Ppn gene expression for all stage 13–16 tissue-stage terms. Scores are indicated either as fold enrichment above random expectation. Terms with high fold enrichments are outlined and colored according to the tissue type. *Bottom*. FISH for Ppn on embryos of different stages. The first two images correspond to the same embryo, where different focal planes have been selected to show expression on the dorsal vessel and visceral muscle. d. Accurate prediction of frequent muscle and midgut co-expression. *Top*. Table showing values for expression predictions in muscle and midgut at late embryos in the 8 genes we performed *in situ*s for muscle expression validation. We bold the scores of genes with more than 10-fold enrichment in either tissue. *Bottom*. FISH images for the indicated genes in stage 13–14 embryos, with focal plane including both somatic muscle and gut. DAPI staining is included (blue signal) to show the shape of the embryo. Scale bar corresponds to 20μm in all images shown.

We started by examining gene expression patterns in two tissues (BDGP *in situ* ontology categories) that had both a large number of genes with high scoring predictions (all predictions above threshold 0.8) and are easy to assess in *in situ* images: brain primordium (stages 11–12) and embryonic muscle system (stages 13 and later). We found that all genes newly predicted to be expressed in theses tissues with no prior annotations were confirmed. For brain primordium, all 5 genes with detectable FISH signals as predicted by FIND were indeed highly expressed in brain at stages 11 or 12 (S1 Table, Fig 3a). For 11 more genes, literature evidence corroborated the FIND predictions (S1 Table). Thus 16/16 tested genes exhibit the FIND- predicted expression pattern for brain primordium. For the embryonic muscle system (stages 13 and later) all 8 genes with detectable FISH signals showed patterns in muscle systems, including in the somatic muscle, visceral muscle and dorsal vessel (the fly embryo heart) (S2 Table, Fig 3b–3d). For 5 more genes, literature evidence again corroborated the FIND predictions (S3 Table). Thus 13/13 tested genes exhibited the FIND-predicted expression pattern for embryonic muscle system. Importantly, in all cases where we have spatio-temporal expression information, the transcript was expressed on the predicted tissue, underscoring the precision of the method.

We have also tested predictions for very specific tissues and organs, such as late embryo Malpighian tubules or salivary glands. We performed FISH for four (Malpighian tubules) and five (salivary glands) genes with more than 20-fold enrichment above background, and in all cases we were able to detect expression on the respective tissues (S5 Fig). Thus, we verified all high confidence predictions that we tested, indicating the utility of using FIND predictions to guide future studies.

To determine how FIND predicts new multi-tissue expression patterns, we compared the predicted spatial-temporal patterns with experimentally measured gene expression across tissue and developmental stages. A particularly interesting example is the gene *Ppn* (*Papilin*). The main cell type where this gene is expressed is the plasmatocyte (Fig 3c), which is consistent with previous observations from Fly-FISH [2]. In addition, we could confirm the previously reported expression in the dorsal vessel [8] and also detected *Ppn* expression in the visceral muscle and somatic muscle primordia (Fig 3c, bottom). Importantly, our model gave all of these tissues high prediction scores for *ppn* expression (Fig 3c, top), showing that complex expression patterns can be accurately predicted from our method.

**Fig 4:**
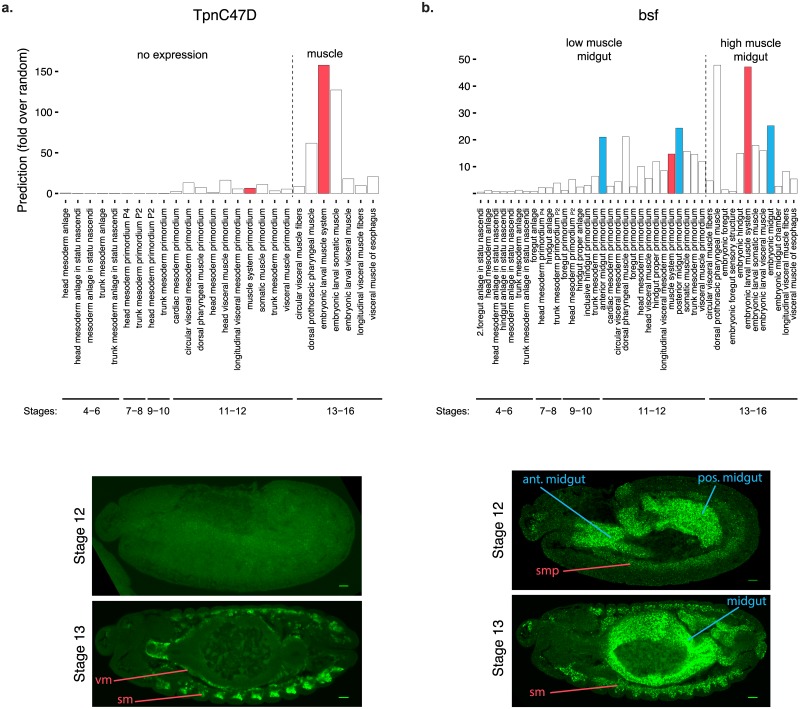
FISH validation of FIND temporal dynamics predictions. a. Expression prediction for TpnC47D up-regulation in muscle. Top. fold enrichment values of the TpnC47D gene for all BDGP terms including either “muscle” or “mesoderm”, ordered from earliest to latest. The terms “Stage 11–12 muscle system primordium” and “Stage 13–16 embryonic larval muscle system”, which represent the same group of structures in different stages, have been colored in red. Bottom. FISH images of the TpnC47D gene expression in stage 12 and stage 13 embryos. b. Prediction of relative changes in expression between muscle and gut for the bsf gene. Top. Same as in a, but also including gut-related terms. Midgut terms for Stage 11–12 and Stage 13–16 BDGP intervals have been colored in blue. Bottom. FISH images of the bsf gene expression in stage 12 and stage 13 embryos.

We next assessed how the variation of gene expression probabilities across stages are related to temporal variation in expression levels during development for specific genes. For example, in the case of *TpnC47D*, the predicted expression scores (Fig 4a) are very high for the term embryonic larval muscle system (stages 13–16) but much lower for the precursor term muscle system primordium (stages 11–12). Indeed, experimental data confirmed this sharp up-regulation of expression, as expression in muscle is only detected from stage 13 and completely absent at earlier stages (Fig 4a). Regulation of *bsf* expression provides another complex and interesting example. *bsf* is predicted to have similar expression level throughout stages 11–16 in midgut but with an increase of ~3 fold in muscle for the same temporal transition. As predicted, the *bsf* gene has a clear pattern of co-expression in the midgut and muscle at stage 13 and later, but when looking at stage 12, the expression in muscle is much lower than in the midgut primordia (Fig 4b).

In conclusion, joint analysis of predictions for multiple tissue-developmental stage terms (either different tissues at a fixed time interval, or across time intervals for one tissue) can be a very valuable tool to guide the investigation of complex spatial-temporal expression patterns for individual genes or groups of genes.

In addition to using spatiotemporal expression pattern predictions to generate hypotheses for individual genes, these predictions can also enable new spatio-temporal specificity analysis of high-throughput experimental data. In case studies below, we showcase two new directions of such applications.

### Case study 1: Using spatio-developmental gene expression predictions for understanding tissue-specificity at transcriptome-scale

High-throughput expression profiling and differential expression analysis are common and important techniques for exploratory analysis of molecular responses to different perturbations. While perturbations such as genetic ablation of a specific gene are expected to affect certain tissues preferentially, this is usually difficult to assess since, for practical reasons, expression profiling experiments are often performed on the whole body or tissue homogenates. FIND’s quantitative score for every tissue and stage for every gene in the genome can detect tissue-specificity signals from whole-organism differential expression experiments, even in the absence of reliable tissue-specific markers. This is particularly useful for tissues with few annotated marker genes. As a proof-of-concept, we used three differential gene expression datasets comparing whole-embryo mutant vs. wild type to predict the tissues most affected by these perturbations independently of any prior knowledge other than FIND predictions (Table 1).

**Table 1:** Identification of relevant tissues from whole-embryo differential expression datasets.

Rank	Mef2 (9-19h)	Twist (4-5h)	Trh (5-16h)
**1**	somatic muscle primordium (stage 11–12)	embryonic larval visceral muscle (stage 13–16)	embryonic salivary gland common duct (stage 13–16)
**2**	embryonic larval visceral muscle (stage 13–16)	dorsal pharyngeal muscle primordium (stage 11–12)	embryonic larval posterior spiracle (tracheal system) (stage 13–16)
**3**	dorsal prothoracic pharyngeal muscle (stage 13–16)	head visceral muscle primordium (stage 11–12)	embryonic salivary gland duct (stage 13–16)
**4**	visceral muscle of esophagus (stage 13–16)	visceral muscle primordium (stage 11–12)	embryonic larval dorsal branch (tracheal system) (stage 13–16)
**5**	embryonic larval muscle system (stage 13–16)	somatic muscle primordium (stage 11–12)	embryonic larval dorsal trunk (tracheal system) (stage 13–16)
**6**	embryonic larval somatic muscle (stage 13–16)	longitudinal visceral mesoderm primordium (stage 11–12)	embryonic salivary gland common duct (stage 13–16)

Top predicted tissue-stages among all 282 tissue-stage categories for three differential expression datasets—mef2 mutant (9-19h), twist mutant (4-5h) and trachealess mutant (5-16h).

For example, *Myocyte enhancer factor 2* (*Mef2)* loss-of-function mutants have defects in myoblast fusion and terminal muscle differentiation [9], consistent with the role of Mef2 as an essential conserved transcriptional regulator of muscle development. We use the transcriptome analysis of this mutant (E-TABM-57) as input for FIND-analysis, and indeed this affected tissue is precisely detected by our computational method: all top six terms (out of 282) predicted to be the most affected in the mutants are muscle tissues (Table 1). Specifically, we rank tissues based on Spearman correlation of the differential gene expression log fold changes and the prediction scores for each of the 282 tissue-stages. The second example is Twist, an upstream regulator of Mef2 that has an earlier and broader role in mesoderm development [10]. *Twist* loss-of-function mutants lead to a block in gastrulation and a loss of mesodermal cells (E-TABM-162). Accordingly, our method predicts the loss of a wide range of mesoderm derivatives in *twist* loss-of-function mutants, and at earlier stages than those predicted for *Mef2* (Table 1). Finally, the third differential expression dataset analyzed a development time-course of *trachealess* (*trh*) mutant versus wildtype whole embryo samples. The *trh* gene codes for an essential transcription factor in the development of the tracheal system [11] (E-GEOD-28780). In agreement with this role, four out of six of the top predictions are tracheal tissue terms. The other two top predictions are related to the salivary gland duct, another major tissue affected by *trh* loss-of-function mutants [11] (Table 1).

In summary, for all three mutants, we are able to make accurate and informative tissue-specificity predictions from experiments performed on whole embryos, without using any prior knowledge of the functions of these genes or phenotypes of their ablation, We therefore expect our tissue expression predictions and methods to be useful for improving detection of tissue signals in other high-throughput experiments where the available known tissue markers are insufficient.

### Case study 2: Identifying potential human disease models in *Drosophila*

While *Drosophila melanogaster* provides a flexible and powerful genetic model for a wide range of human diseases, it is not always clear which tissue is the best choice for modeling a certain human disease. This is especially relevant when a direct tissue equivalent is absent in the fly. To address this challenge, we examined FIND-predicted tissue-stage patterns in the fly embryo for human disease genes. Specifically, we identified fly orthologs of human disease gene from OMIM database, using a functional mapping approach which identifies gene that are not only similar in sequence, but also share similar roles in pathways in human and fly [12]. Then, embryonic tissues were ranked based on their enrichment of genes related to each disease, thus prioritizing fly tissues by their relevance to human disease.

**Fig 5:**
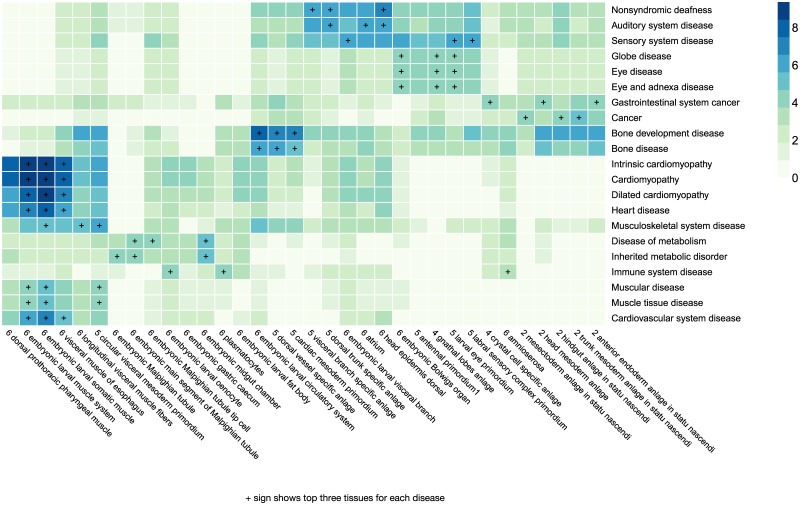
Prioritization of fly developmental tissues and stages for modeling human disease. Heatmap showing false discovery rate (-log10 FDR) for enrichment of human OMIM disease gene functional orthologs in each tissue-stage combination, based on FIND predictions (p-value is computed based on PAGE). The number before tissue names indicate stages (1: stage 1–3, 2: stage 4–5, 3: stage 6–8, 4: stage 9–10, 5: stage 11–12, 6: stage 13–16).

Among the diseases with most significant tissue enrichment, muscle diseases and cardiomyotrophy was mapped to somatic muscles (Fig 5). Despite the distinct morphology of fly eyes, larval eye primordium is selected as the best tissue for modeling for eye diseases (Fig 5). Indeed, fly eye is known to share key developmental biology with mammals, for example, the *Drosophila eyeless* and its mammal ortholog *Pax6* are both essential to eye development [13]. Auditory system diseases are best mapped to the dorsal head epidermis, as well as sensory complex (Fig 5). Even though flies lack a skeletal system, bone diseases are mapped best to mesoderm primordium tissues, suggesting that at least their early development during mesoderm stage may be modeled (Fig 5). In conclusion, the results show that genome-wide predictions of tissue-stage expression can assist in identifying and developing new human disease models.

### Discussion

By integrating diverse expression and chromatin profiles with *in situ* tissue-specific gene standards, we provided accurate predictions of spatio-developmental expression patterns for all annotated genes, both coding and non-coding. Enabling our approach is a cell lineage-aware computational method, SIND, that uses a unified spatial-temporal transcription probabilistic graphical model to integrate predictions for developmentally related tissues informed by tissue developmental ontology. We have both computationally and experimentally verified the potential of these predictions, confirming the accuracy and predictive power of our method.

The whole-genome coverage and quantitative resolution of our approach thus can not only provide researchers with predicted spatio-temporal expression patterns for over 4,000 genes that lack experimental evidence, but also power their data analyses, including detection of tissue-specificity signals in non-tissue resolved (e.g. whole embryo) expression datasets and identification of most relevant tissues for study of human disease.

To facilitate the exploration of our spatio-developmental gene expression predictions and downstream analyses, we provide a user-friendly web interface (http://find.princeton.edu). This web tool allows for querying tissue-gene predictions, discovering genes with similar tissue-specific expression patterns and predicting tissue-stage specificity from a user specified list of genes (e.g. differential gene expression after a perturbation experiment).

It is important to note that our approach currently covers only embryonic developmental stages due to limitations in availability of in-situ data at other stages for training; this could be addressed by applying this approach to additional temporal stages as training data becomes available. Furthermore, the ability to predict gene expression patterns in highly-specific cell types depends on the input data compendiums being informative of the gene co-expression patterns in these cell types and the availability of known expressed genes from these specific cell types as labels. Single cell data can thus potentially contribute to training both as input data and as labels in the future, although such an approach will need to address noisiness and the challenges of robust definition of cell types from single cell data. Lastly, of course, even with high accuracy and coverage, the resources that we provide are still computational predictions, and should be treated as such. Their power is in enabling analysis and directing follow-up experiments.

Finally, we note that this resource is built upon, and complementary, to current genomic data, and will continually benefit from a progressively growing amounts of *in situ* tissue specificity standards and RNA-seq and chromatin profiling data, as well as integrating new types of genomic features like sequence features or new experimental techniques. This flexibility is an important property for such a dynamic field as functional genomics.

## Methods

### Data

The data for training the *Drosophila melanogaster* spatio-developmental expression prediction model are obtained and processed as follows. *Drosophila melanogaster* raw microarray data for the two major platforms GPL1322 and GPL72 were downloaded from NCBI Gene Expression Omnibus at May 2014 and processed with the *GEO2R* package. Missing values in microarrays were imputed with KNNImpute [14]. Drosophila melanogaster RNA-seq and ChIP-seq raw reads data were downloaded from NCBI Sequence Read Archive (SRA) and aligned to BDGP5 fly genome assembly using bowtie2 with local alignment mode [15]. RNA-seq alignment data was summarized by RPKM using BDGP v5.78 gene annotation (and lncRNA annotation from [16]). Non-transcriptomic sequencing alignment data was summarized by RPKM within ± 500bp region to TSS. modENCODE RNA-seq dataset was downloaded from [16]. The datasets used are listed in S3 File. All sequencing samples were log-transformed after adding a pseudo-count. The pseudo-count was determined as half of the value of the lowest non-zero gene for each sample. All samples were standardized to mean 0 and variance 1.

Tissue- and developmental stage- specific expression standards were obtained from BDGP *in situ* database (insitu.fruitfly.org) and FlyFISH database (fly-fish.ccbr.utoronto.ca) on May 2014. Genes included in both microarray platforms were used. Since BDGP *in situ* annotation are more comprehensive in fine tissue structures, we use BDGP as the main standards while using FlyFISH as assistance standards that were only used to train first-stage classifiers. The subset of genes mapped to microarray platforms were used, with a holdout set strictly not used in any steps of algorithm design and the decision of hyperparameters for evaluation.

Our Drosophila embryonic development tissue developmental ontology is modified from [1] with a few changes adapted from flybase tissue developmental ontology FBdv [17]. The ontology we used is provided in S1 File.

### Structured in-silico nano-dissection for spatio-developmental gene expression prediction

Our machine learning algorithm uses a cell-lineage aware approach to predict gene expression with spatio-developmental specificity based on whole-genome scale expression data. The cell lineage information is critical for specific tissues and stages that can benefit from considering developmental context including its precursor and descendant tissues.

Our model takes *in situ* gene expression annotations and functional genomics data as input and performs prediction in the stages. In the first stage, one individual classifier was trained for each individual tissue-stage term. Specifically, for each tissue-stage term, a linear structural SVM model was trained to predict *in situ* annotations (whether a gene is annotated to a tissue-stage) from functional genomic datasets including transcriptome profiling data (microarray and RNA-seq) and chromatin profiling data (ChIP-seq). The model was trained using the SVMperfer tool in the Sleipnir library [18].

In the second stage, we use a conditional random field classifier, which integrates individual tissue-stage predictions using learned dependencies of gene expression between tissue-stage terms. The conditional random field model represents each tissue-stage as a node and cell-lineage relations as edges connecting the nodes. Tissue-stage nodes are connected with the following rules:

1. If they are in a developmental precursor-and-descendent relationship and in the same or adjacent stages.
2. If they are directly connected in the tissue developmental ontology by the “part of “relationship and in the same or adjacent stages.
3. If they are the same tissue type in adjacent stages.
4. The “no staining” terms were connected to all other terms in the same stage (because of the expected negative dependencies).

The CRF model that directly provides probability output for each gene and each tissue is formulated as the following

$$P(Y|X)=\frac{1}{Z}e^{\sum_{m}^{}{(W}_{m}X{+\alpha_{m})Y}_{m}+\sum_{(m,n)\in G_{\text{ontology}}}^{}J_{mn}Y_{m}Y_{n}}$$

P(Y│X) describes the conditional probability model. The conditional probability is determined by a term that describes dependencies on first-stage individual classifiers output ∑_*m*_(*W*_*m*_*X* + *α*_*m*_)*Y*_*m*_ and a cell lineage dependency term $\sum_{(m,n)\in G_{\text{ontology}}}J_{mn}Y_{m}Y_{n}$. Note that if the second term does not exist the model reduces to multiple logistic regression models. Specifically, Y is the vector of indicator variables for expression in tissue-stages, where *Y*_*m*_ = 1 indicates expression in the *m*-th tissue-stage and *Y*_*m*_ = 0 indicates no-expression in the tissue-stage indexed by *m*. *X* represents a matrix containing all SVM predictions from the first stage classifiers, in which a column corresponds to a classifier. The model is also illustrated by a diagram in S6 Fig.

*W*, *J*, *a* are estimated from the input (first stage predictions and *in situ* annotations). Specifically in the first term *W*_*m*_ represents weight the *m*-th tissue-stage classifier from the first stage, which is analogous to coefficients of a logistic regression model and *α*_*m*_ is the intercept. We allow a tissue-stage to be informed by predictions for any tissue-stage classifiers in the first term, which integrate expression signals over expression predictions for multiple tissue-stage-specific classifiers based on annotations from both BDGP *in situ* and FlyFISH.

In the second term, *J*_*mn*_ represents interaction between tissue-stage *m* and *n* and only edges in the tissue developmental ontology are allowed to be non-zero. G_ontology_ represent the ontology based graph structure which is a set of edges built with rules described above. Prediction for a tissue-stage can either increase or decrease probability of a connected tissue-stage term depending on the estimated dependency scores *J*. *Z* is the normalizing constant which ensures the sum of probabilities equals 1, and it does not need to be explicitly computed in our learning algorithm.

The model can be trained by minimizing the L1 Penalized likelihood ∑log P(Y│X) + *λ*|*J*|. The L1 regularization *λ*|*J*| is used to avoid overfitting to tissue-stage dependencies by encouraging *J* to be 0 if it contributes little to the likelihood. |*J*| represents the L1 norm of interactions J which equals sum of absolute values of all entries of *J*. *λ* is the L1-regularization parameter.

To prevent overfitting to the *in situ* annotations, for the training of the second stage model, all predictions from the first stage were made on holdout genes with 5 fold cross-validation. Specifically, for each fold, 4/5 of the genes were used for training and the trained model was used to predict the rest 1/5 holdout genes. The concatenated predictions on the holdout set for each fold, which covers all the training set genes, were used as input to the second stage classifier. As the concatenated predictions were all predicted using models that have not seen the gene before, it minimizes the bias due to overfitting in the second stage.

### Optimization of the structured in-silico nano-dissection model parameters

Next, we describe the optimization algorithm for minimizing the objective function. Computing the exact gradient of the CRF likelihood over the ontology-based graph is intractable since the graph is not tree or chain structured. Approximation inference algorithms, such as pseudo-likelihood-based method is biased but computationally efficient, while Markov chain Monte Carlo (MCMC) sampling-based inference methods converge to the exact gradient in the limit of large sample size, but is computationally expensive to obtain low variance gradient estimate.

To address both speed and accuracy, we choose to use a hybrid of both. We first obtain a fast approximation of parameters by optimizing a modified pseudo-likelihood objective,

$$\sum_{i}^{}\sum_{m}^{}\text{log}\;P(Y_{m}^{\left(i\right)}|X^{\left(i\right)},Y_{-m}^{\left(i\right)})+\lambda |J|=\sum_{i}^{}\sum_{m}^{}\frac{e^{\sum_{m}^{}{(W}_{m}X{+\alpha_{m})Y}_{m}+\sum_{(m,n)\in G_{\text{ontology}}}J_{mn}Y_{m}Y_{n}}}{e^{\sum_{m}^{}{(W}_{m}X{+\alpha_{m})Y}_{m}+\sum_{(m,n)\in G_{\text{ontology}}}J_{mn}Y_{m}Y_{n}}+1}+\lambda |J|$$

for which the gradient can be computed efficiently because it does not involve computing *Z* which is intractable, and initialize the second step optimization which optimizes the true objective with the estimators from first step. The L1 regularized optimization problem was solved with Projected scaled sub-gradient (Gafni-Bertsekas variant) as implemented in (https://www.cs.ubc.ca/~schmidtm/Software/thesis.html).

For the second step, we estimate the stochastic gradient of the model objective by Gibbs sampling and employ stochastic gradient descent with momentum for optimization. For Gibbs sampling based gradient estimation, we first rewrite the likelihood as

$$P(Y|X)=\frac{1}{Z}e^{\sum_{m}^{}f_{m}(X)Y_{m}+\sum_{(m,n)\in G_{\text{ontology}}}J_{mn}Y_{m}Y_{n}}$$

To compute the stochastic gradient for the log-likelihood when conditioned on X, we used the fact (ignored writing down conditioning on X for simplicity)

$$\frac{\partial log\;(P)}{\partial f_{m}}=\bar{Y_{m}}-E(Y_{m}),\frac{\partial log\;(P)}{\partial J_{mn}}=\bar{Y_{m}Y_{n}}-E(Y_{m}Y_{n})$$

where expectations E(Y_m_) and E(Y_m_Y_n_) are estimated by MCMC sampling. For each iteration, we run a separate Markov chain for each gene and average the stochastic gradients obtained from each chain over all genes. To address efficiency and burn-in time of MCMC, we initialize MCMC chains from the last sample of the previous iteration (also called persistent MCMC sampling), and for each iteration, we only run one sweep of Gibbs sampling which sample each node exactly once and use the single sample Y* to compute estimator $E(Y_{m})=Y_{m}^{*}$ and $E(Y_{m}Y_{n})=Y_{m}^{*}Y_{n}^{*}$. The Markov chains for all genes are parallelized for speeding up.

For each iteration, we increment model parameters based on stochastic gradient descent with specified step size. We use a learning rate annealing scheme of 100,000 iterations of learning rate 1e-6, followed by 50,000 iterations of 1e-7 and 50,000 iterations of 1e-8. Additionally, a momentum term was used for stabilizing the update and improving convergence. L1 regularization was applied to control overfitting. We set momentum to 0.9 and L1-regularization parameter to 8.

After the model is trained, for prediction from the second-stage CRF model we ran 80,000 rounds of Gibbs sampling and average over the samples. For genes with *in situ* annotations, we predict their expression with 5 fold cross-validation.

### Mapping human disease to fly embryonic development tissue-stages

Human disease gene sets were obtained from OMIM. We selected the functional ortholog of human genes in *Drosophila melanogaster* using functional knowledge transfer (FKT) method with p-value cutoff 0.1 [12]. For each fly gene the lowest p-value human ortholog was chosen, then each tissue-stage expression prediction profile is mapped to human genes accordingly (a total of 4937 fly genes were mapped with above cutoff confidence). Statistical significance of gene set enrichment in each mapped tissue-stage expression prediction profile was computed with parametric analysis of gene set enrichment (PAGE) method [19].

### Fluorescent *in situ* hybridization

Fluorescent *in situ* hybridizations were performed as previously described [20]. Briefly, we used cDNA clones from the DGRC collections DGC1 and 2 [21] for preparing digoxigenin-labelled or fluorescein-labeled RNA probes, except for the CG14688 gene for which we use a previously reported cDNA template [22] and CG34284 for which we amplified a cDNA fragment. In this case, we performed Biozym Red HS Master Mix (Biozym) using as template total cDNA obtained from mixed stage of D. melanogaster wt embryos (an overnight embryo collection) and the primers GTCGGTCATTGAAAGTTTCC and CCGAACATATTCCAAATCTG). The amplified fragment (495 pb) was cloned into pGEM-T easy (Promega) and the positive clones were verified by sequencing. Probes were detected with peroxidase-conjugated antibodies (Roche) and developed using the TSA-plus Tyramide fluorescence system (Perkin Elmer) for fluorescein or Cy3 fluorophore deposition.

Imaging was performed in a Zeiss LSM 780 confocal microscope, using a Plan-Apochromat 20x/0.8 objective. For fluorescein detection, we used the 488nm wavelength of a multiline Argon laser, a MSB 488/561 dichroic mirror and a spectral detector set for a 500-600nm detection range. For Cy3 detection, samples were excited with a DPSS laser at. For DAPI detection, samples were excited with a diode at 405nm and the emission fluorescence was detected with a MBS405 dichroic mirror. Other acquisition parameters, such as PMT gain and offset, were set independently for each probe, dependent on the signal. After the acquisition, images were processed using ImageJ 1.46r [23]. Although a Z-stack was routinely acquired, a single focal plane was manually selected for visualization, except for the images of Malpighian tubules and salivary glands, where a Z-projection was used as indicated. Post-acquisition processing consisted on cropping and orienting single embryos and adjusting minimum and maximum intensity values for maximizing contrast.

### Code and data availability

The code is available at https://github.com/FunctionLab/find_scripts. The gene expression prediction dataset is available at http://find.princeton.edu/predictions/download/ or https://doi.org/10.5281/zenodo.3408411.

## Supporting information

S1 Fig Comparison of different tissue-stage classifier performances. Classifiers trained using a single data type, ChIP or RNA-seq, from a large collaborative project, modENCODE, was compared to classifiers using all public data from NCBI GEO for a single data type, and classifiers using all public NCBI GEO data sets and all data types. Integrating prediction utilizing tissue relationships information by ontology-CRF method further improved prediction performances. The left panel compares AUCs for each tissue-stage category with and without using ontology-CRF integration. For every input type, the cross-validation performances for all tissue-stage categories measured by area under ROC curves (AUC) were shown with boxplot.
(TIFF)

S2 Fig Global structure of predicted tissue-stage transcriptomes by developmental stages. The two dimensional projections of high-dimensional gene expression probability predictions were obtained with linear discriminant analysis (LDA) to best separate the stages. Each point corresponds to a tissue-stage, and the color represent the developmental stage.
(TIFF)

S3 Fig Global structure of predicted tissue-stage transcriptomes visualized by multidimensional scaling. The two dimensional projections of high-dimensional gene expression probability predictions were obtained with multidimensional scaling (MDS). The first two principal coordinates approximately correspond to variation across stages (a) and the third and fourth principal coordinates approximately correspond to variation across tissue types (b). Each point corresponds to a tissue-stage, and the color represents developmental stage in (a) and tissue type in (b).
(TIFF)

S4 Fig Consistent high performance of tissue-stage specificity predictions for genes of varying levels of expression complexity. The gene expression tissue specificity prediction performances for all genes are measured by AUROC based on cross-validation. The distribution of AUROCs for genes with different gene expression annotation complexities were compared, showing better prediction performance for low complexity genes but retaining good performance even for high complexity genes.
(TIFF)

S5 Fig Validation of FIND predictions in specific late-embryo organs. a. Four genes with more than 20-fold enrichment above background in the term "6.embryonic Malpighian tubule" were selected for FISH on late embryos (> stage 13). Images show Z-projections of 2–3 focal planes from entire embryos, with the signal for the specific gene probe in red. mt: Malpighian tubules; hg: hindgut; mg: midgut. b. Five genes with more than 20-fold enrichment above background in the term "6.embryonic salivary gland" were selected for FISH on late embryos (> stage 13). Images show Z-projections of 2–3 focal planes from zoomed anterior sections of embryos. Signal for the specific gene probe is shown in red and signal for the salivary gland marker Gld is shown in green. For clarity, in some cases we pointed at the salivary gland cells with arrowheads based on the Gld signal. DAPI staining is included (blue signal) to show the shape of the embryo. Scale bar corresponds to 20μm in all images shown.
(TIFF)

S6 Fig Schematic overview of the SIND algorithm. The structured in-silico nano-dissection(SIND) algorithm make prediction based on dependencies of each tissue-stage term on SVM prediction, represented by X, and network connectivities between each term and the terms connected in the developmental ontology, represented by J. The SVM prediction dependency parameters W, *α* and network connectivity parameter J are learned during training on tissue-stage labels Y.
(TIFF)

S1 Table Validation of brain expression predictions. List of the 17 genes selected to validate the expression prediction for the BDGP term “5.brain primordium”. Since 5^th^ BDGP category corresponds to stages 11 and 12, we inform the range of modENCODE RNA-seq expression values for time points 6-8h and 8-10h, and we inform a positive result when expression in the predicted tissue is seen in stage 11 or 12 embryos (last column). Evidence from literature encompasses all references listed in FlyBase for each gene. Evidence from hold-out *in situ* data includes FlyFISH expression patterns as well as BDGP *in situs* that have not been included in the training set for any reason. In all cases, we report “yes” if the reported expression includes expression in the brain during stages 11 or 12. Related evidence is also stated.
(DOCX)

S2 Table Validation of muscle expression predictions. List of the 17 genes selected to validate the expression prediction for the BDGP term “6.embryonic larval muscle system”. Since 6^th^ BDGP category corresponds to stages 13 and later, we inform the range of modENCODE RNA-seq expression values for time points from 10-12h to the end of embryogenesis. Similarly, we inform a positive result when expression in the predicted tissue is seen in stage 13 or later (last column). Evidence from literature encompasses all references listed in FlyBase for each gene. Evidence from hold-out *in situ* data includes FlyFISH expression patterns as well as BDGP *in situs* that have not been included in the training set for any reason. In all cases, we report “yes” if the reported expression includes expression in the brain during stages 13 or later. Related or contradictory evidence (in red) is also stated.
(DOCX)

S3 Table Summaries of experimental validation of genes outside training set. Table summarizing both the results of our FISH experiments (first three rows) and the whole evidence available to support the expression of the genes in the predicted tissue (last four rows).
(DOCX)

S1 File The tissue-developmental ontology for Drosophila melanogaster embryonic development. (PDF)

S2 File Cross-validation performance of our predictions for all tissue-stages. (TXT)

S3 File List of all genome-wide transcriptome and chromatin data sets used in this study. (XLSX)
