# Supplementary figures and images for: Accurate genome-wide predictions of spatio-temporal gene expression during embryonic development
Source: PLoS Genet. 2019 Sep 25;15(9):e1008382. doi: 10.1371/journal.pgen.1008382 (PMC6779412; doi:10.1371/journal.pgen.1008382)

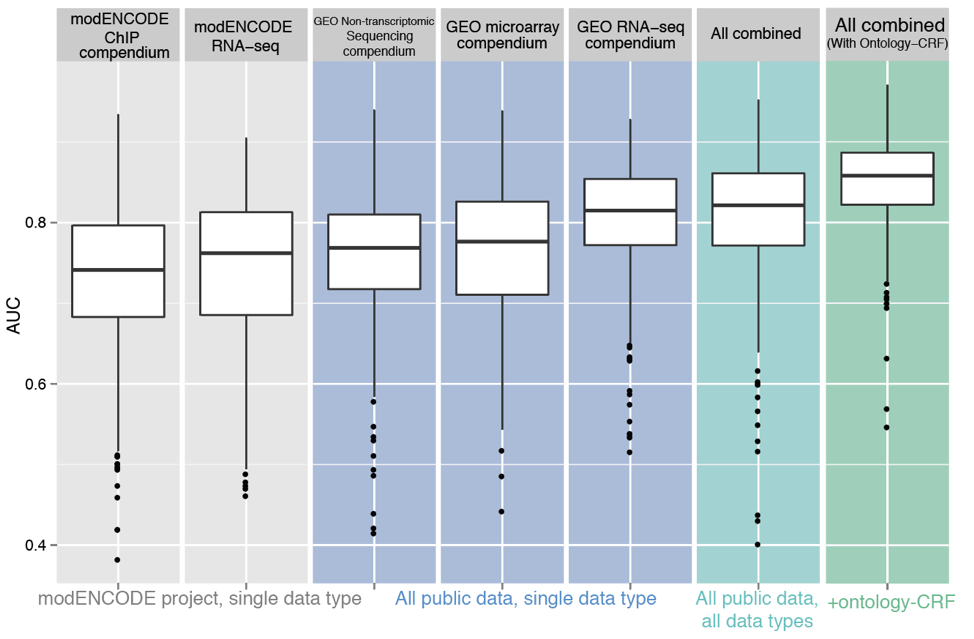

Supplement: S1 Fig — Classifiers trained using a single data type, ChIP or RNA-seq, from a large collaborative project, modENCODE, was compared to classifiers using all public data from NCBI GEO for a single data type, and classifiers using all public NCBI GEO data sets and all data types. Integrating prediction utilizing tissue relationships information by ontology-CRF method further improved prediction performances. The left panel compares AUCs for each tissue-stage category with and without using ontology-CRF integration. For every input type, the cross-validation performances for all tissue-stage categories measured by area under ROC curves (AUC) were shown with boxplot. (TIFF) [file pgen.1008382.s001.tiff]

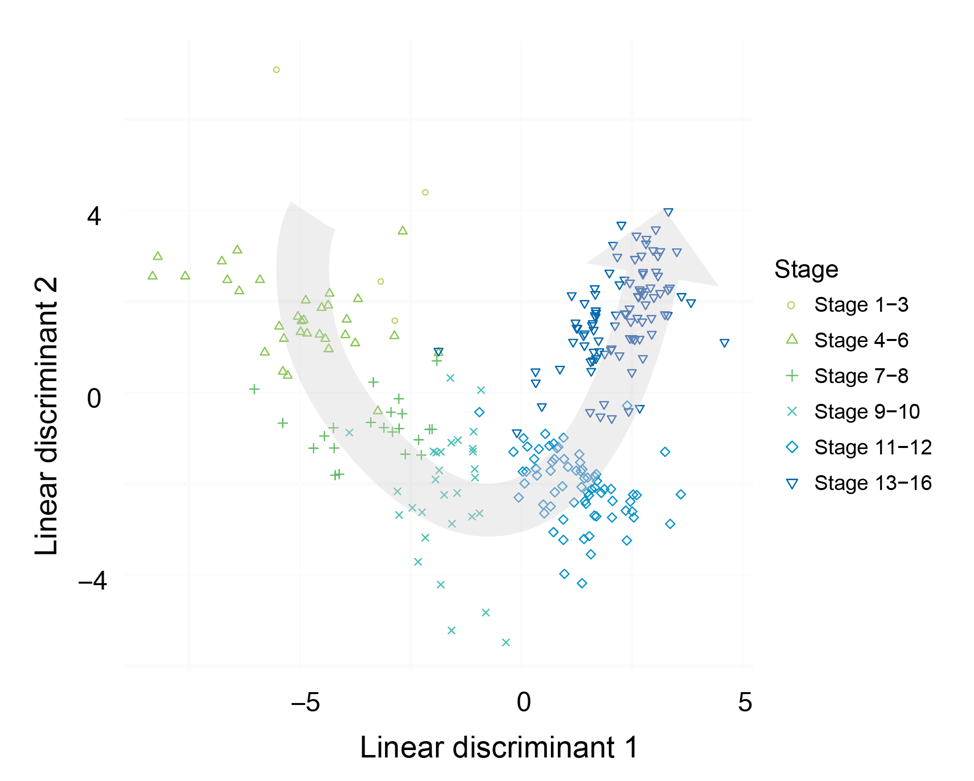

Supplement: S2 Fig — The two dimensional projections of high-dimensional gene expression probability predictions were obtained with linear discriminant analysis (LDA) to best separate the stages. Each point corresponds to a tissue-stage, and the color represent the developmental stage. (TIFF) [file pgen.1008382.s002.tiff]

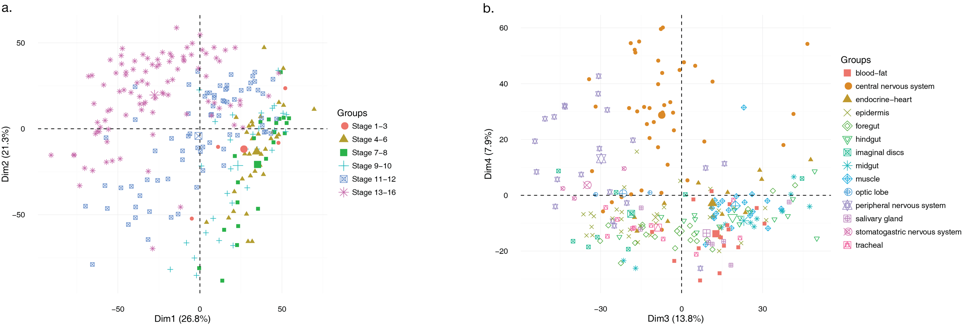

Supplement: S3 Fig — The two dimensional projections of high-dimensional gene expression probability predictions were obtained with multidimensional scaling (MDS). The first two principal coordinates approximately correspond to variation across stages (a) and the third and fourth principal coordinates approximately correspond to variation across tissue types (b). Each point corresponds to a tissue-stage, and the color represents developmental stage in (a) and tissue type in (b). (TIFF) [file pgen.1008382.s003.tiff]

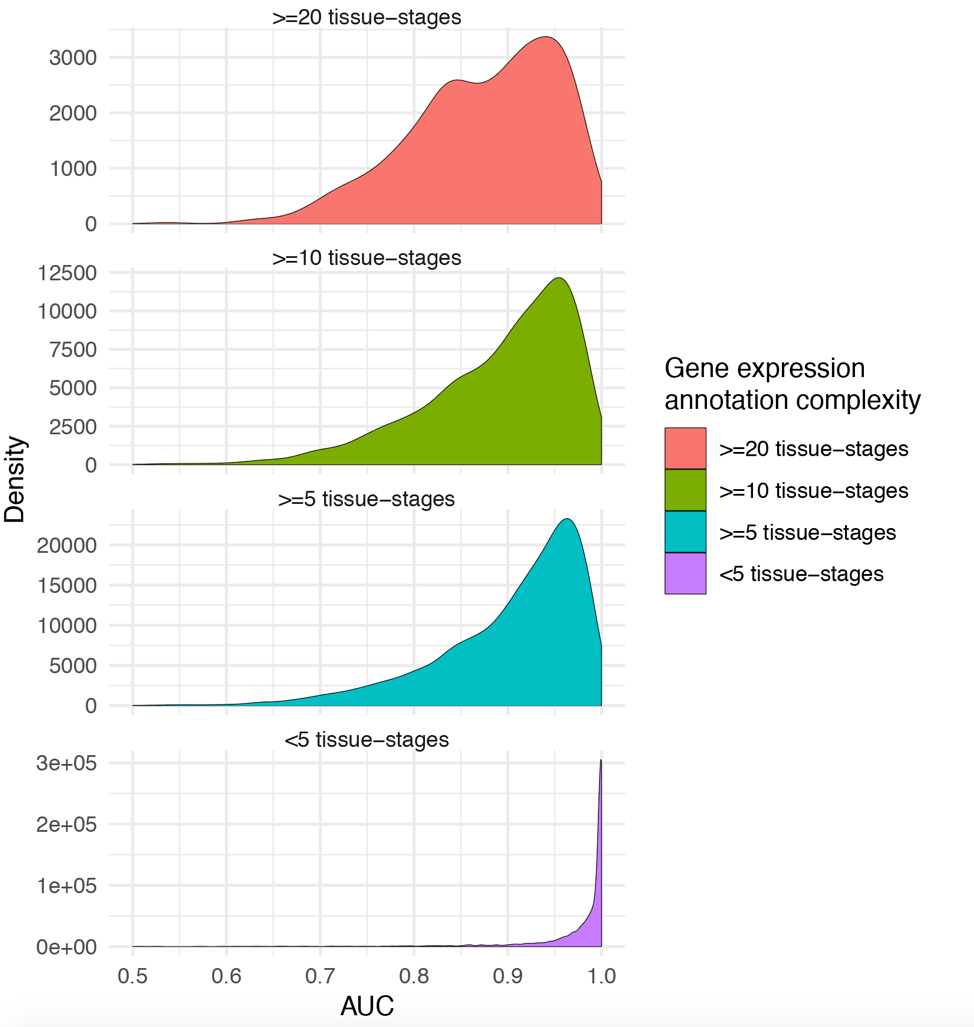

Supplement: S4 Fig — The gene expression tissue specificity prediction performances for all genes are measured by AUROC based on cross-validation. The distribution of AUROCs for genes with different gene expression annotation complexities were compared, showing better prediction performance for low complexity genes but retaining good performance even for high complexity genes. (TIFF) [file pgen.1008382.s004.tiff]

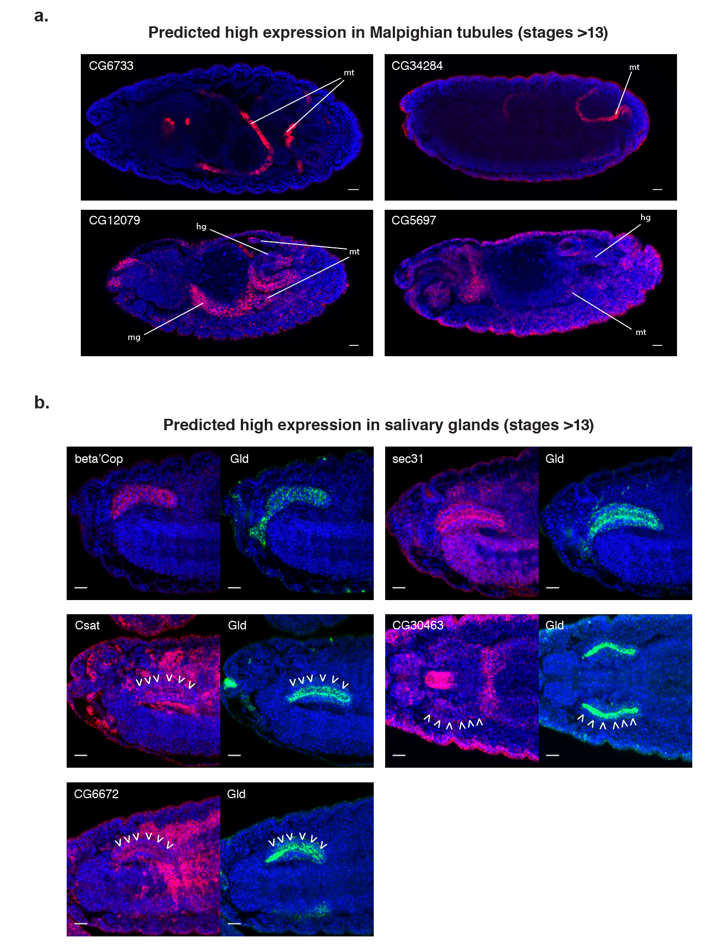

Supplement: S5 Fig — a. Four genes with more than 20-fold enrichment above background in the term "6.embryonic Malpighian tubule" were selected for FISH on late embryos (> stage 13). Images show Z-projections of 2–3 focal planes from entire embryos, with the signal for the specific gene probe in red. mt: Malpighian tubules; hg: hindgut; mg: midgut. b. Five genes with more than 20-fold enrichment above background in the term "6.embryonic salivary gland" were selected for FISH on late embryos (> stage 13). Images show Z-projections of 2–3 focal planes from zoomed anterior sections of embryos. Signal for the specific gene probe is shown in red and signal for the salivary gland marker Gld is shown in green. For clarity, in some cases we pointed at the salivary gland cells with arrowheads based on the Gld signal. DAPI staining is included (blue signal) to show the shape of the embryo. Scale bar corresponds to 20μm in all images shown. (TIFF) [file pgen.1008382.s005.tiff]

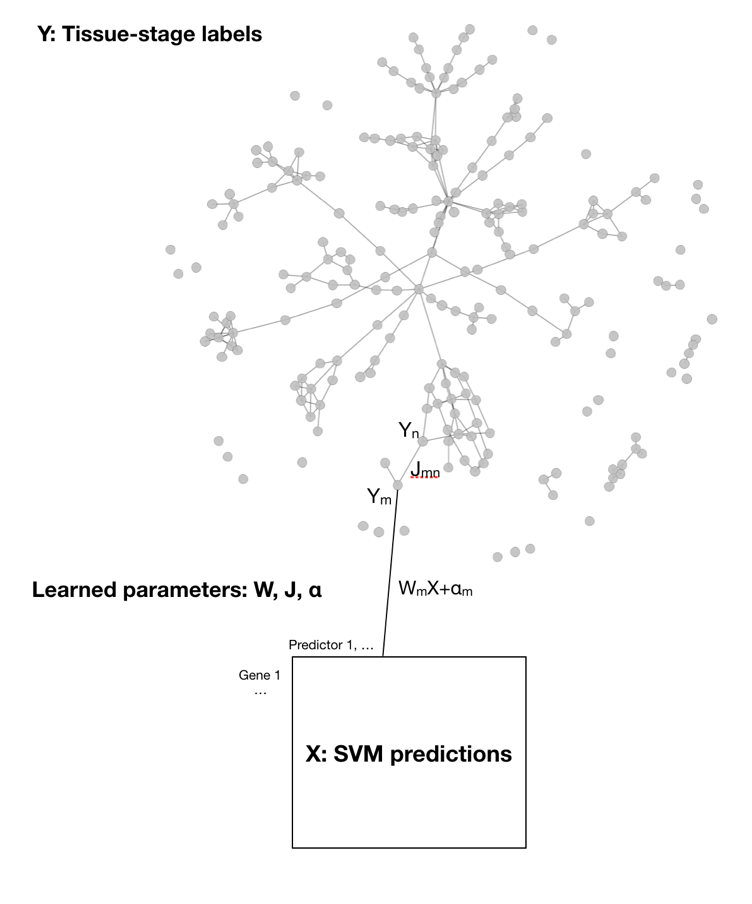

Supplement: S6 Fig — The structured in-silico nano-dissection(SIND) algorithm make prediction based on dependencies of each tissue-stage term on SVM prediction, represented by X, and network connectivities between each term and the terms connected in the developmental ontology, represented by J. The SVM prediction dependency parameters W, α and network connectivity parameter J are learned during training on tissue-stage labels Y. (TIFF) [file pgen.1008382.s006.tiff]

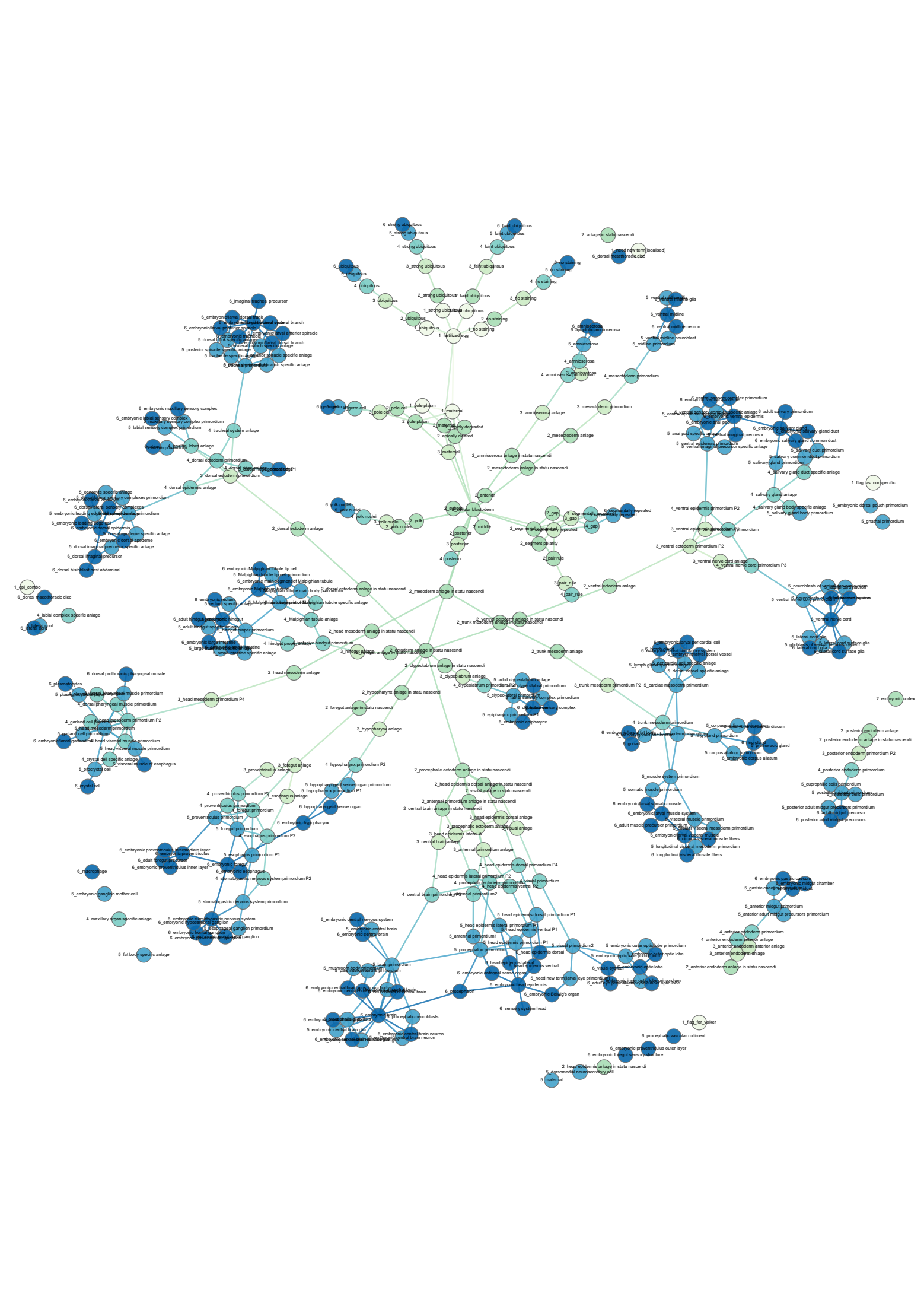

Supplement: S1 File — (PDF) [file pgen.1008382.s010.pdf]
