# Supplementary material for: Accurate genome-wide predictions of spatio-temporal gene expression during embryonic development
Source: PLoS Genet. 2019 Sep 25;15(9):e1008382. doi: 10.1371/journal.pgen.1008382 (PMC6779412; doi:10.1371/journal.pgen.1008382)
Supplement: S1 Table — List of the 17 genes selected to validate the expression prediction for the BDGP term “5.brain primordium”. Since 5th BDGP category corresponds to stages 11 and 12, we inform the range of modENCODE RNA-seq expression values for time points 6-8h and 8-10h, and we inform a positive result when expression in the predicted tissue is seen in stage 11 or 12 embryos (last column). Evidence from literature encompasses all references listed in FlyBase for each gene. Evidence from hold-out in situ data includes FlyFISH expression patterns as well as BDGP in situs that have not been included in the training set for any reason. In all cases, we report “yes” if the reported expression includes expression in the brain during stages 11 or 12. Related evidence is also stated. (DOCX) [file pgen.1008382.s007.docx]

**S1 Table. Validation of brain expression predictions.**

List of the 17 genes selected to validate the expression prediction for the BDGP term “5.brain primordium”. Since 5^th^ BDGP category corresponds to stages 11 and 12, we inform the range of modENCODE RNA-seq expression values for time points 6-8h and 8-10h, and we inform a positive result when expression in the predicted tissue is seen in stage 11 or 12 embryos (last column). Evidence from literature encompasses all references listed in FlyBase for each gene. Evidence from hold-out *in situ* data includes FlyFISH expression patterns as well as BDGP *in situs* that have not been included in the training set for any reason. In all cases, we report “yes” if the reported expression includes expression in the brain during stages 11 or 12. Related evidence is also stated.

| **Gene ID** | **Gene name** | **Expression level WE 6-10h (RPKM)** | **Literature-based evidence** | **Hold-out *in situ* evidence** | **With related GO terms** | **Results from new FISH experiments - Expression at stages 11-12** |
| --- | --- | --- | --- | --- | --- | --- |
| **31394** | **pon** | **>50** | neuroblasts |  | yes | peripheral nervous system, VNC and brain |
| **34842** | **spel1** | **9-17** |  |  |  | brain and ventral nerve cord |
| **35649** | **Incenp** | **>50** |  |  | yes | brain and ventral nerve cord |
| **47121** | **dup** | **>50** |  |  |  | brain, VNC and periph. nerv. system, gut |
| **36967** | **CG6520** | **>50** |  |  |  | brain and ventral nerve cord |
| **34250** | **Cks30A** | **35-65** |  |  |  | no FISH signal detected |
| **39826** | **fax** | **>50** | yes | ubiquitous | yes |  |
| **318559** | **spdo** | **>50** | yes |  |  |  |
| **31603** | **Mcm6** | **>50** | yes |  |  |  |
| **36703** | **unc-5** | **>50** | yes |  | yes |  |
| **43157** | **m4** | **>50** | yes |  | yes |  |
| **35225** | **Top2** | **>50** |  | yes |  |  |
| **3772396** | **seq** | **>50** |  | yes | yes |  |
| **37482** | **LBR** | **>50** |  | yes |  |  |
| **39654** | **Msh6** | **14-30** |  | yes |  |  |
| **40232** | **polo** | **>50** |  | yes |  |  |
| **43228** | **ball** | **>50** |  | yes | yes |  |
|  |  |  |  |  |  | *VNC: ventral nerve cord* |
