# Supplementary material for: Accurate genome-wide predictions of spatio-temporal gene expression during embryonic development
Source: PLoS Genet. 2019 Sep 25;15(9):e1008382. doi: 10.1371/journal.pgen.1008382 (PMC6779412; doi:10.1371/journal.pgen.1008382)
Supplement: S2 Table — List of the 17 genes selected to validate the expression prediction for the BDGP term “6.embryonic larval muscle system”. Since 6th BDGP category corresponds to stages 13 and later, we inform the range of modENCODE RNA-seq expression values for time points from 10-12h to the end of embryogenesis. Similarly, we inform a positive result when expression in the predicted tissue is seen in stage 13 or later (last column). Evidence from literature encompasses all references listed in FlyBase for each gene. Evidence from hold-out in situ data includes FlyFISH expression patterns as well as BDGP in situs that have not been included in the training set for any reason. In all cases, we report “yes” if the reported expression includes expression in the brain during stages 13 or later. Related or contradictory evidence (in red) is also stated. (DOCX) [file pgen.1008382.s008.docx]

**S2 Table. Validation of muscle expression predictions.**

List of the 17 genes selected to validate the expression prediction for the BDGP term “6.embryonic larval muscle system”. Since 6^th^ BDGP category corresponds to stages 13 and later, we inform the range of modENCODE RNA-seq expression values for time points from 10-12h to the end of embryogenesis. Similarly, we inform a positive result when expression in the predicted tissue is seen in stage 13 or later (last column). Evidence from literature encompasses all references listed in FlyBase for each gene. Evidence from hold-out *in situ* data includes FlyFISH expression patterns as well as BDGP *in situs* that have not been included in the training set for any reason. In all cases, we report “yes” if the reported expression includes expression in the brain during stages 13 or later. Related or contradictory evidence (in red) is also stated.

| **Gene ID** | **Gene name** | **Expression level 10-24h (RPKM)** | **Literature-based evidence** | **Hold-out *in situ* evidence** | **With related GO terms** | **Results from new FISH experiments - Expression at stages 13-16** |
| --- | --- | --- | --- | --- | --- | --- |
| **41275** | **CG14688** | **23-71** | related (mesoderm) |  |  | somatic muscle (up to stage 13) |
| **43872** | **Ppn** | **>50** | related (mesoderm, heart) |  |  | plasmatocytes, visceral muscle, heart |
| **34950** | **Ca-alpha1D** | **3-9** | yes | against (salivary glands) | yes | visceral and somatic muscle, nerv. system |
| **35100** | **bsf** | **24-42** |  |  |  | muscle, gut |
| **36195** | **TpnC47D** | **>50** |  |  |  | somatic and visceral muscle |
| **44438** | **EfTuM** | **>50** |  |  |  | muscle, gut |
| **43102** | **CG5028** | **>50** |  |  |  | muscle, gut |
| **31811** | **l(1)G0020** | **9-31** |  |  |  | muscle, gut |
| **35007** | **Mhc** | **>50** | yes |  | yes |  |
| **34068** | **LanB1** | **>50** | related (mesoderm) |  |  | no FISH signal detected |
| **37419** | **Glycogenin** | **15-36** |  | yes |  |  |
| **39414** | **Smyd4** | **5-17** |  | yes |  |  |
| **44010** | **Pgm** | **>50** |  | yes |  |  |
| **34021** | **CG5261** | **>50** |  |  |  | no FISH signal detected |
| **36617** | **RpI1** | **11-34** |  |  |  | no FISH signal detected |
| **39143** | **ATPsyn-b** | **>50** |  |  |  | no FISH signal detected |
| **53565** | **bor** | **>50** |  |  |  | no FISH signal detected |
