# Supplementary material for: Accurate genome-wide predictions of spatio-temporal gene expression during embryonic development
Source: PLoS Genet. 2019 Sep 25;15(9):e1008382. doi: 10.1371/journal.pgen.1008382 (PMC6779412; doi:10.1371/journal.pgen.1008382)
Supplement: S3 Table — Table summarizing both the results of our FISH experiments (first three rows) and the whole evidence available to support the expression of the genes in the predicted tissue (last four rows). (DOCX) [file pgen.1008382.s009.docx]

**S3 Table. Summaries of experimental validation of genes outside training set.**

Table summarizing both the results of our FISH experiments (first three rows) and the whole evidence available to support the expression of the genes in the predicted tissue (last four rows).

| **Summary of evidence for 'Stage 11-12 Brain primordium'** | |
| --- | --- |
| # Tested with FISH in this study | 6 |
| With detectable FISH signal | 5/6 |
| With expression in the predicted tissue | 5/5 |
| Confirmed by any direct evidence | 16/17 |
| With direct or indirect evidence | 16/17 |
| Negative result (present in tissue other than predicted) | 0/17 |
| No evidence of expression pattern | 1/17 |
| **Summary of evidence for 'Stage 13-16 Embryonic muscle system'** | |
| # Tested with FISH in this study | 13 |
| With detectable FISH signal | 8/13 |
| With expression in the predicted tissue | 8/8 |
| Confirmed by any direct evidence | 12/17 |
| With direct or indirect evidence | 13/17 |
| Negative result (present in tissue other than predicted) | 0/17 |
| No evidence of expression pattern | 4/17 |
